# COVID-19 severity and vaccine effectiveness in Malawi: A test-negative case-control study

**DOI:** 10.4102/jphia.v16i1.758

**Published:** 2025-04-11

**Authors:** Clara Sambani, Victor Chikwapulo, Regina Mankhamba, Tonny Muwonge, Mavuto Thomas, Baxter Salatiel, Edna Mandala, Leah Mbabazi, Suzan Nakasendwa, Rodgers R. Ayebare, Collins Mitambo, Matthew Kagoli, Mabvuto Chiwaula, Dzinkambani Kambalame, Triza Chirwa, Liness Chinyamunyamu, Tamrat Shaweno, Nebiyu Dereje, Tajudeen Raji, Francis Kakooza, Mosoka P. Fallah, Evelyn C. Banda, Abigail Kazembe, Mitch Matoga

**Affiliations:** 1 Public Health Institute of Malawi, Lilongwe, Malawi; 2 World Health Organization, Malawi Office, Lilongwe, Malawi; 3 Global Health Security Department, Infectious Diseases Institute, Makerere University, Kampala, Uganda; 4 Health Education Division, Ministry of Health, Lilongwe, Malawi; 5 School of Maternal, Neonatal and Reproductive Health, Kamuzu University of Health Sciences, Lilongwe, Malawi; 6 Department of Global Health Security, Infectious Diseases institute, Makerere University, Kampala, Uganda; 7 Dowa District Hospital, Ministry of Health, Lilongwe, Malawi; 8 Directorate of Research and Innovation, Africa Centres for Disease Control and Prevention, Addis Ababa, Ethiopia; 9 Department of Global Health Security, Infectious Diseases Institute, Makerere University, Lilongwe, Uganda; 10 African Forum for Research and Education in Health, Kumasi, Ghana; 11 University of North Carolina Project, Lilongwe, Malawi

**Keywords:** COVID-19, vaccine, effectiveness, risk, severity, hospitalisation, Malawi

## Abstract

**Background:**

COVID-19 vaccines were administered globally, and Malawi commenced vaccination on 11 March 2021.

**Aim:**

This study assessed the real-world effectiveness of COVID-19 vaccines and factors associated with disease severity and mortality in Malawi.

**Setting:**

A facility-based case-control study within the largest referral hospitals.

**Methods:**

Cases (COVID-19 positive) were matched 1:1 with controls based on age, sex and testing date. Interviews via phone focused on COVID-19 testing and vaccination, underlying conditions and disease outcomes. Analysed using STATA 17, the exposure of interest was vaccination status. For vaccine effectiveness (VE), conditional logistic regression modelling was used, while disease severity and management were analysed using binary logistic regression.

**Results:**

The unvaccinated were at 53.3%, and 35.8% were fully vaccinated and/or received a booster. The VE among the fully and partially vaccinated was 10% (95% CI: –26.2, 35.81) and 31.8% (95% CI: –9.91, 57.72), respectively compared to the unvaccinated. Most of the COVID-19 patients (87.8%) were not hospitalised. Underlying chronic conditions and a previous positive COVID-19 test were associated with severe disease (aOR: 3.54, 95% CI: 1.65, 7.61 and aOR: 2.73, 95% CI: 1.13, 7.61, respectively); however, these odds were not different by vaccination status.

**Conclusion:**

The VE was low and severe disease was linked with chronic illnesses and previous positive COVID-19 tests. Efforts to promote vaccination through education and access should be enhanced, particularly for those with underlying chronic conditions.

**Contribution:**

The findings can inform strategies on prioritisation for disease vaccination and improving patient outcomes.

## Introduction

COVID-19, caused by the severe acute respiratory syndrome coronavirus 2 (SARS-CoV-2), has been a disease of global health importance because of its rapid spread and massive impact on lives and livelihoods all over the world.^1,2^ On 30 January 2020, the World Health Organization (WHO) officially classified the COVID-19 epidemic as a Public Health Emergency of International Concern (PHEIC).^3^ Following large concerted public health responses across the globe, the trend of the disease changed over time, and the WHO rescinded its PHEIC classification on 05 May 2023.^4^ As of 06 December 2023, the pandemic had globally resulted in approximately 772 138 818 reported cases and 6 985 964 confirmed fatalities, while Malawi had registered approximately 89 089 cases and 2686 deaths.^5^ As part of the response to this pandemic, vaccines were developed expeditiously and administered globally. The vaccines were proven to effectively reduce transmission, morbidity and mortality, and mass vaccination, a proven strategy for protecting susceptible individuals in the population, was the solution to bring an end to the pandemic and safeguard lives and livelihoods.^6,7^

The COVID-19 waves of SARS-CoV-2 in Malawi, in reference to nationally reported COVID-19 figures, indicate that wave 1 was from April 2020 to October 2020; wave 2 was from November 2020 to March 2021; wave 3 ran from April 2021 to August 2021 and wave 4 was from December 2021 to March 2022.^8^ Malawi introduced the COVID-19 vaccination campaign on 11 March 2021.^9^ The vaccines approved by the Pharmacy Medicines and Poisons Authority (PMRA) for emergency use included Pfizer BioNTech, AstraZeneca (AZ) and Johnson & Johnson (J&J).^10^ As of 06 December 2023, approximately 30.2% of the eligible population (individuals who were 18 years and above for AZ and J&J; and those 12 years and above for Pfizer)^9,11^ had been fully vaccinated, and only 8.1% had received a booster dose.^12^ This low coverage persists despite other government efforts such as implementing the COVID-19 Vaccine Express (CVE) strategy, the ‘Vaccinate My Village’ strategy and routine COVID-19 vaccination, where vaccination was rolled out to communities and hard-to-reach areas with sufficient supplies.^9^ The low uptake of the COVID-19 vaccine is apparent.

Clinical trials are the gold standard for establishing the efficacy and safety of vaccines.^13,14^ Despite their importance in establishing vaccine performance, clinical trials do not provide data on the performance of vaccines in the real world. Coronavirus disease 2019 vaccines showed satisfactory results during rapid clinical trials and equally received regulatory rapid approvals owing to the serious public health emergency the disease posed. However, one of the main barriers to the uptake of vaccines is the lack of confidence in the effectiveness of the vaccines by the general public because of their rapid development, highlighting the need to understand their real-world effectiveness.^15,16^ Evaluating real-world COVID-19 vaccine performance is critical for understanding the risks and benefits of vaccination programmes.^13^ Some of the factors that affect real-world vaccine effectiveness (VE) include prior infection, and transportation and storage of vaccines. In addition, the people vaccinated in clinical trials are often young and healthy and, therefore, different from those who receive vaccines in the real world.^17,18^ We therefore aimed to evaluate the COVID-19 VE in the Malawian population and the risk of hospitalisation and/or developing severe COVID-19 disease.

## Research methods and design

### Study design

This was a test-negative case-control study design. We used COVID-19 testing and admission records of community-dwelling individuals eligible for COVID-19 vaccination who presented themselves to a testing or treatment centre meeting the criteria for a patient under investigation for SARS-CoV-2 infection in Malawi. Patients under investigation were those who presented to the health care worker with at least one of the following symptoms: fever, cough, shortness of breath and any other symptom based on the COVID-19 variant and country-specific criteria for suspected disease. Coronavirus disease 2019 cases were defined as patients under investigation for COVID-19 with a positive laboratory-confirmed diagnosis of SARS-CoV-2 infection, and controls were those who tested negative for SARS-CoV-2 using polymerase chain reaction (PCR) or rapid antigen diagnostic test.

### Study setting

The identification of study participants as cases and controls was made from the national COVID-19 testing database (March 2021 to September 2022), which was during the third and fourth COVID-19 waves. The database had COVID-19 testing records of individuals who presented to testing centres or isolation units and met the case definition for COVID-19. We used testing and admission records from Mzuzu Central Hospital (Northern region), Kamuzu Central Hospital (Central region) and Queen Elizabeth Central Hospital (Southern region), the largest referral hospitals in Malawi. The implementation areas were selected based on being urban areas, were considered a hotspot for the COVID-19 epidemic, had a high population density based on the overall country population estimate and had a high COVID-19 positivity rate. Being referral hospitals, with a high density and hotspot for COVID-19, there were multiple testing sites within the facility. The referred cases from surrounding districts were also being tested for COVID-19.

### Study population and sampling strategy

Using the national COVID-19 testing database, participants whose SARS-CoV-2 was positive were matched with participants whose SARS-CoV-2 was negative. The matching age range was plus or minus 5 years, males were matched to males and females were matched to females, and within the same month of COVID-19 testing. The participants were matched and selected from the database using randomly generated numbers in Stata version 17. To ensure a low dropout rate among the matched pairs, one negative participant was matched to three positive participants because the database had more SARS-CoV-2 positives than negatives. For each completed interview with a negative participant, we contacted each of the paired positives until we had a successful interview among the three. Among the three positive participants, one participant was selected randomly without replacement until a positive participant was found.

### Sample size

Sample size calculations were based on a generic protocol designed for 12 countries and assumed a 60% VE, 80% confidence interval and 10% adjustment for non-response. The ratio of controls to cases was set at 1:1. The estimated sample size was 1000 participants for each country, implying 500 controls and 500 cases in Malawi.

### Data collection methods

Data were collected using a standardised structured questionnaire. Between 11 April 2023 and 17 August 2023, research assistants administered the survey questionnaire via phone using contact details from the testing and admission database. A computerised data collection tool programmed using Research Electronic Data Capture (REDCap) software was used to capture the data from the participants recruited in the study. Using mobile gadgets, the data were entered directly into Case Report Forms (CRFs) in REDCap software. The questionnaire comprised variables including demographics, COVID-19 testing information, COVID-19 vaccination information, underlying conditions and outcome of the COVID-19 disease, including hospitalisation and the severity. Other data, such as the date of vaccination were obtained from the national vaccination database.

Vaccination status was defined as ‘fully vaccinated/boosted’ when an individual had received two doses of a two-dose vaccine or one dose of a single-dose vaccine and/or received a booster dose; ‘partially vaccinated’ when an individual had received only one dose of a two-dose vaccine at least 14 days before they presented for a test and ‘unvaccinated’ when an individual had not received a single dose of any of the available vaccines at the time of testing.

Primary outcome was having COVID-19 positive test (case) or negative (control), while the secondary outcomes included: (1) COVID-19 disease management categorised as home-based care or hospitalised, (2) COVID-19 severity categorised as mild, moderate (hospitalised but did not need oxygen and not admitted to intensive care unit [ICU]) or severe (hospitalised and needed oxygen and/or admitted to ICU) and (3) COVID-19 outcome categorised as recovered, or died. All secondary outcomes were only assessed among participants with a positive test for COVID-19 (500 cases).

### Data analysis

The demographic characteristics were analysed and presented as frequencies, percentages and graphically. To determine the VE, conditional logistic regression was used to estimate the risk (through odds ratios) of testing positive for SARS-CoV-2 infection, with vaccination status as the primary exposure of interest. Vaccine effectiveness was estimated as one minus the odds ratio (OR); VE = (1 – OR) * 100. Multivariable conditional logistic regression was used to estimate odds ratios, comparing the odds of vaccination among cases with the odds of vaccination among controls while adjusting for covariates. Confounding was assessed by comparing crude and adjusted VE for each baseline characteristic. The risks of COVID-19-related hospitalisation and COVID-19 severity were determined through odds ratios using binary logistic regression and ordinal logistic regression, respectively.

Pooled data from Mzuzu, Lilongwe and Blantyre provided sufficient power to estimate VE at the national level. During model building for secondary outcomes, variables with a *p*-value of ≤ 0.25 from the bivariate analysis and those that were deemed important in the study were included in the multivariable model. During the modelling process, interactions were tested using the likelihood ratio test and included in the model at a 5% significance level. Data were analysed in Stata software version 17.

### Ethical considerations

Ethical approval was obtained from the College of Medicine Research Ethics Committee (protocol no.: 01/23/3945). The authors also obtained verbal informed consent from all participants, who agreed to be part of the study. The research team ensured confidentiality and privacy for participants. The participants were free to withdraw from the study at any point. Only the study team had access to the data that were collected in the study.

## Results

### Demographics, underlying conditions, vaccination status and vaccine effectiveness

**FIGURE 1:**
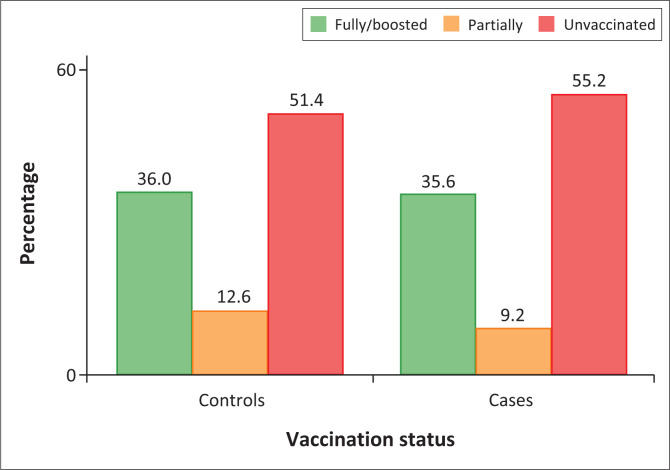
Vaccination status among study participants in Malawi at the time of COVID-19 testing.

Overall, more than half of the study participants were unvaccinated, that is, 533 (53.3%), and 358 (35.8%) were fully vaccinated and/or boosted. Among controls, 257 (51.4%) were unvaccinated, compared to 276 (55.2%) among cases (Figure 1).

As shown in Table 1, the majority of study participants were male (*n* = 570, 56.9%), and most participants were aged between 25 years and 45 years (*n* = 669, 66.9%). Among the study participants, 800 (80%) were tested using point-of-care (rapid), and about a quarter (*n* = 268, 26.8%) had a history of another prior positive COVID-19 test. The majority of the vaccinated participants had received the AstraZeneca vaccine on both the first (*n* = 293, 62.7%) and second (*n* = 210, 89.4%) doses. We observed that slightly more controls (48.6%, *n* = 243) were vaccinated compared to cases (44.8%, *n* = 224). Among all participants, 176 (17.6%) had at least one underlying chronic condition including hypertension, asthma, diabetes and human immunodeficiency virus (HIV). The history of current alcohol consumption was more frequent than smoking (23.6% versus 3.3%), but both were slightly higher among the controls (Table 1).

**TABLE 1:** Demographic and vaccination characteristics of participants.

Demographic characteristics	Frequency (*N* = 1000)		Cases (*n* = 500)		Controls (*n* = 500)	
	*n*	%	*n*	%	*n*	%
**Age range (years)**						
18–24	126	12.6	58	11.6	68	13.6
25–35	425	42.5	212	42.4	213	42.6
36–45	244	24.4	125	25.0	119	23.8
46–55	162	16.2	83	16.6	79	15.8
> 55	43	4.3	22	4.4	21	4.2
**Sex**						
Female	430	43.0	215	43.0	215	43.0
Male	570	57.0	285	57.0	285	57.0
**Occupation**						
Healthcare worker	111	11.1	55	10.1	56	11.2
Other	889	88.9	445	89.0	444	88.8
**Test type**						
PCR	200	20.0	88	17.6	112	22.4
Point-of-care	800	80.0	412	82.4	388	77.6
**Testing frequency (among those with a history of past positive tests) (*n* = 268)**						
Once	177	66.0	40	63.5	137	66.8
Twice	63	23.5	17	27.0	46	22.4
Thrice	16	6.0	1	1.6	15	7.3
More than thrice	12	4.5	5	7.9	7	3.4
**COVID-19 vaccination status**						
Fully Vaccinated/boosted	358	35.8	178	35.6	180	36.0
Partially vaccinated	109	10.9	46	9.2	63	12.6
Unvaccinated	533	53.3	276	55.2	257	51.4
**First dose**						
Johnson and Johnson	136	29.1	63	28.1	73	30.0
Sinopharm	1	0.2	0	0.0	1	0.4
AstraZeneca	293	62.7	146	65.2	147	60.4
Pfizer/BioNTech	37	7.9	15	6.7	22	9.1
**Second dose**						
Johnson and Johnson	20	8.5	8	6.8	12	10.3
AstraZeneca	210	89.4	108	91.5	102	87.2
Pfizer/BioNTech	5	2.1	2	1.7	3	2.6
**Underlying chronic condition**						
No	824	82.4	399	79.8	425	85.0
Yes	176	17.6	101	20.2	75	15.0
**Smoking status**						
Never	925	92.5	471	94.2	454	90.8
Stopped	39	3.9	17	3.4	22	4.4
Current	33	3.3	11	2.2	22	4.4
Unknown	3	0.3	1	0.2	2	0.4
**Alcohol consumption**						
Never	693	69.3	350	70.0	343	68.6
Stopped	64	6.4	34	6.8	30	6.0
Current	236	23.6	112	22.4	124	24.8
Unknown	7	0.7	4	0.8	3	0.6
**Pregnancy**						
Yes	57	13.2	34	15.7	23	10.7
No	352	81.5	173	80.1	179	82.9

Note: Age in years: Frequency: Median = 34, IQR = 43; cases: Median = 28, IQR = 42; controls: Median = 28, IQR = 42.

PCR, polymerase chain reaction; COVID-19, coronavirus disease 2019; IQR, interquartile range.

The primary VE among the fully vaccinated participants was approximately 10% compared to unvaccinated participants (95% CI: –26.2, 35.81). The VE was observed as –23.3% among fully vaccinated participants with at least one chronic condition compared to those without any. For participants who were vaccinated with at least one dose of the COVID-19 vaccines (fully + partially vaccinated), compared to the unvaccinated, the VE was 15.6% (95% CI: –16.3, 38.8), whereas for the partially vaccinated participants, the VE was 31.84% (95% CI: –9.91, 57.72) against the unvaccinated; but this was not statistically significant (Table 2).

**TABLE 2:** Vaccine effectiveness among the study participants.

Vaccination status	OR		VE	
	%	95% CI	%	95% CI
Fully vaccinated versus unvaccinated	0.90	0.64, 1.26	9.99	−26.20, 35.81
Partially vaccinated versus unvaccinated	0.68	0.42, 1.10	31.84	−9.91, 57.72
Fully + partially vaccinated versus unvaccinated	0.84	0.61, 1.16	15.60	−16.3, 38.8
Fully vaccinated versus unvaccinated, stratified by having at least one chronic condition	1.23	0.75, 2.02	−23.34	−102.44, 24.86

VE, vaccine effectiveness; OR, odds ratio.

### COVID-19 vaccination and the risk of severe COVID-19 disease and hospitalisation

Generally, among the COVID-19 cases, most (87.8%, *n* = 439) did not require hospitalisation for their COVID-19 infection and were managed at home. Among those who were hospitalised, about half (54.8%, *n* = 17) were hospitalised for less than a week. A total of 19 (61.3%) needed oxygen, while 13 (41.9%) were admitted to the ICU. The case fatality rate was very low (< 1%). There was one reported death among the cases who was an unvaccinated hypertensive male, aged 58 years old (Table 3).

**TABLE 3:** Hospitalisation and management of the COVID-19 cases.

Clinical outcome	Frequency (*n*)	%
**Type of management (*n* = 500)**		
Home-based care	439	87.8
Hospitalisation	31	6.2
Missing	30	6.0
**Duration of hospitalisation (*n* = 31)**		
< 1 week	17	54.8
1–2 weeks	10	32.3
> 2 weeks	4	12.9
**Needed oxygen (*n* = 31)**		
Yes	19	61.3
No	12	38.7
**Admitted to intensive care unit/high dependency unit (*n* = 31)**		
Yes	13	41.9
No	18	58.1
**COVID-19 outcome (*n* = 500)**		
Recovered	469	93.8
Died	1	0.2
Missing	30	6.0

COVID-19, coronavirus disease 2019.

### Risk factors associated with COVID-19 severity and hospitalisation among COVID-19 cases

Having at least one chronic condition (adjusted odds ratio [aOR]: 3.54, 95% CI: 1.65, 7.61) and positive history of COVID-19 (aOR: 2.73, 95% CI: 1.13, 6.63) were statistically associated with higher chances of having moderate and then severe COVID-19 (Table 4).

**TABLE 4:** Association between demographic characteristics and COVID-19 severity.

Characteristics	COVID-19 severity						Crude OR	95% CI	Adjusted OR	95% CI
	Mild (*n* = 432)		Moderate (*n* = 11)		Severe (*n* = 27)					
	*n*	%	*n*	%	*n*	%				
**Vaccination status**										
Fully vaccinated boosted	161	37.4	3	27.3	8	33.0	Ref	Ref	Ref	Ref
Partially vaccinated	41	9.5	1	9.1	3	11.1	1.31	0.40, 4.27	1.14	0.32, 3.98
Unvaccinated	230	53.2	7	63.6	15	55.6	1.28	0.61, 2.65	1.45	0.67, 3.13
**Age categories (years)**										
18–24	48	11.1	1	9.1	2	7.4	Ref	Ref	Ref	Ref
25–35	191	44.2	4	36.4	8	29.6	1.01	0.27, 3.70	0.90	0.23, 3.47
36–45	106	24.5	4	36.4	7	25.9	1.65	0.44, 6.18	1.31	0.33, 5.14
46–55	69	16.0	1	9.1	8	29.6	2.14	0.55, 8.31	1.38	0.33, 5.79
> 55	18	4.2	1	9.1	2	7.4	2.65	0.49, 14.30	1.26	0.20, 7.73
**Gender**										
Female	190	44.0	4	36.4	8	29.6	Ref	Ref	Ref	Ref
Male	242	56.0	7	63.6	19	70.4	1.71	0.84, 3.47	1.77	0.83, 3.78
**Underlying chronic condition**										
No	355	82.2	6	54.6	15	55.6	Ref	Ref	Ref	Ref
Yes	77	17.8	5	45.5	12	44.4	3.71	1.87, 7.35	3.54	1.65, 7.61
**Occupation**										
Other	48	11.1	0	0.0	2	7.4	Ref	Ref	-	-
Health care worker	384	88.9	11	100.0	25	92.6	0.45	0.11, 1.93	-	-
**Tested positive for COVID-19 before**										
No	384	88.9	10	90.9	20	66.7	Ref	Ref	Ref	Ref
Yes	45	10.4	1	9.1	7	25.9	2.35	1.01, 5.42	2.73	1.13, 6.63
Unknown	3	0.7	0	0.0	0	0.0	-	-	-	-
**Smoking status**										
Never smoked	411	95.1	10	90.9	24	88.9	Ref	Ref	Ref	Ref
Stopped	14	3.2	1	9.1	1	3.7	1.67	0.37, 7.64	1.00	0.16, 6.04
Currently smoking	7	1.6	0	0.0	2	7.4	3.73	0.74, 18.69	4.03	0.71, 22.90
**Alcohol consumption**										
Never drank alcohol	305	70.6	7	63.6	18	66.7	Ref	Ref	Ref	Ref
Stopped	26	6.0	1	9.1	4	14.8	2.38	0.84, 6.71	1.62	0.47, 5.55
Currently drinking	97	22.5	3	27.3	5	18.5	1.00	0.44, 2.28	0.84	0.33, 2.15
Unknown	4	0.9	0	0.0	0	0.0	-	-	-	-
**Pregnancy**										
No	157	82.6	4	100.0	5	62.5	Ref	Ref	-	-
Yes	25	13.2	0	0.0	2	25.0	1.45	0.30, 7.12	-	-

Ref, reference category; COVID-19, coronavirus disease 2019; OR, odds ratio; CI, confidence interval.

We observed a statistically significant association between hospitalisation and underlying chronic conditions, as those with a chronic condition were more likely to be hospitalised (aOR: 5.11 95% CI: 2.21, 11.81).

Participants with older age (46 years and above) were more likely to be hospitalised compared to those below 25 years, although this association was not statistically significant (Table 5).

**TABLE 5:** Association between demographic characteristics and COVID-19 disease management.

Characteristics	COVID-19 management				Crude OR	95% CI	Adjusted OR	95% CI
	Home-based (*n* = 439)		Hospitalised (*n* = 31)					
	*n*	%	*n*	%				
**Vaccination status**								
Fully vaccinated/boosted	163	37.1	10	32.3	Ref	Ref	Ref	Ref
Partially vaccinated	41	9.3	4	12.9	1.59	0.47, 5.33	1.44	0.40, 5.24
Unvaccinated	235	53.5	17	54.8	1.18	0.53, 2.64	1.38	0.58, 3.27
**Age categories (years)**								
18–24	48	10.9	3	9.7	Ref	Ref	Ref	Ref
25–35	192	43.7	11	35.5	0.92	0.25, 3.42	0.82	0.21, 3.29
36–45	110	25.1	7	22.6	1.02	0.25, 4.11	0.70	0.16, 3.03
46–55	71	16.2	7	22.6	1.58	0.39, 6.40	0.87	0.19, 3.94
> 55	18	4.1	3	9.7	2.67	0.49, 14.44	1.03	0.16, 6.62
**Gender**								
Female	192	43.7	10	32.3	Ref	Ref	Ref	Ref
Male	247	56.3	21	67.7	1.63	0.75, 3.55	1.71	0.74, 3.93
**Underlying chronic condition**								
No	361	82.2	15	48.4	Ref	Ref	Ref	Ref
Yes	78	17.8	16	51.6	4.94	2.34, 10.41	5.11	2.21, 11.81
**Occupation**								
Other	391	89.1	29	93.6	Ref	Ref	-	-
Health care worker	48	10.9	2	6.5	0.56	0.13, 2.43	-	-
**Tested positive for COVID-19 before**								
No	389	88.6	25	80.7	Ref	Ref	Ref	Ref
Yes	47	10.7	6	19.4	1.99	0.78, 5.09	2.18	0.80, 5.90
Unknown	3	0.7	0	0.0	-	-	-	-
**Smoking status**								
Never smoked	418	95.2	27	87.1	Ref	Ref	Ref	Ref
Stopped	14	3.2	2	6.5	2.21	0.48, 10.23	1.48	0.23, 9.57
Currently smoking	7	1.6	2	6.5	4.42	0.88, 22.32	4.97	0.82, 30.02
**Alcohol consumption**								
Never drank alcohol	310	70.6	20	64.5	Ref	Ref	Ref	Ref
Stopped	27	6.2	4	12.9	2.30	0.73, 7.20	1.22	0.31, 4.76
Currently drinking	98	22.3	7	22.6	1.11	0.45, 2.70	0.89	0.32, 2.50
Unknown	4	0.9	0	0.0	-	-	-	-
**Pregnancy**								
No	158	82.3	8	80.0	-	-	-	-
Yes	26	13.5	1	10.0	-	-	-	-

Ref, reference category; COVID-19, coronavirus disease 2019; OR, odds ratio; CI, confidence interval.

## Discussion

In this case-control study, we evaluated the uptake of COVID-19 vaccine among the participants, how the disease manifested among them in terms of severity and hospitalisation and assessed any associated factors of these outcomes. We also demonstrated the VE of COVID-19 vaccines among community-dwelling individuals in urban, high-burden areas of Malawi. The VE among the fully vaccinated was at 10%. Also, SARS-CoV-2-positive participants with underlying conditions were associated with a higher risk of severe disease and hospitalisation.

In our study, surprisingly, being fully vaccinated resulted in lesser protective efficacy against COVID-19 infection compared to being partially vaccinated; however, this difference was not statistically significant. The VE for the fully vaccinated compared to unvaccinated was 10%, fully and partially vaccinated compared to unvaccinated was 15.6% and partially vaccinated compared to unvaccinated was 31.8%, but these were not statistically significant. These findings might have been because of the fact that Malawi initiated vaccination very late and people might have sustained some natural immunity^9,19,20^ and our study design was also unable to ascertain the time difference between vaccination and COVID-19 test. However, an observational study conducted at one of the biggest hospitals in southern Malawi showed that there was no difference in the mortality of patients between the vaccinated and unvaccinated.^8^ Contrally to our findings, a retrospective study in Saudi Arabia showed a VE of around 68.1% for Pfizer and AstraZeneca, and other studies have also shown higher VE between 83.0% and 99.5%.^21,22,23^ These higher VE results could be because of differences in the type of vaccines being assessed as well as the study designs. In addition, COVID-19 vaccination has proven to reduce the risk of severe COVID-19 infection within populations. A real-world study in Morocco found that fewer individuals who received a vaccine were hospitalised with severe or critical COVID-19 unlike the unvaccinated (0.65% vs. 0.06%, *p* < 0.001), and no individuals who received booster doses were hospitalised for severe or critical COVID-19.^24^ The United States (US) Centers for Disease Control and Prevention (CDC) also indicated that the ICUs within the lowest vaccinated states have had more patients than the highest vaccination sites. It is important to note that even vaccinated individuals can still get infected with COVID-19.^25,26^

Similar to data from many settings, the COVID-19 vaccine uptake in our study was low. The majority of our study population was unvaccinated (53.3%). In addition, those who were fully vaccinated with or without a booster dose were at 35.8%. Almost half of our participants who tested negative for SARS-CoV-2 were unvaccinated (51.4%), while among the SARS-CoV-2-positive participants, 35.6% were vaccinated. Another study conducted in Malawi showed that 61.0% of participants were unvaccinated and only 33.0% were up-to-date on COVID-19 vaccination,^27^ which is consistent with our findings, while the Malawi Extended Programme of Immunisation reported around 30.0% COVID-19 vaccine coverage around the study period, further substantiating our finding. The comparability of our results with the national data contributes to the generalisability of our findings.

In this study, almost 90% of the participants had mild COVID-19 disease and were treated at home. This outcome is generally the same globally, as most cases were treated at home. The WHO recommended that individuals with mild symptoms of COVID-19 should be managed at home, provided they had the necessary conditions for self-isolation and access to medical advice if needed.^28^ During pandemic preparedness for communicable diseases, it is, therefore, essential to support home-based care programmes in caring for infectious diseases. This highlights the need to develop the capacity of community-based health care workers to manage infectious diseases to avoid the community spread of such diseases.^29^

We found that having an underlying chronic condition increased the risk of severe COVID-19 infection and hospitalisation (aOR: 3.54 and 5.11, respectively). We also found that having tested positive in the last 6 months also increased the risk of severity. This result is similar to other studies which indicate that chronic disease and being tested for COVID-19 in the past 6 months weaken the immune system possibly because of long covid effects like immune dysregulation and dysfunction.^30,31^ On the contrary, a systematic review indicated that getting reinfection lowers the risk of severe illness.^32^ It is important, therefore, to intensify vaccination campaigns, including awareness among the Malawian population with underlying chronic conditions. One plausible strategy could be the integration of vaccination into routine facility clinics for chronic diseases.

Because this was a retrospective study, information was subjected to recall bias as the requested information on vaccination could have been dated months or years prior, and there was also no documented proof of vaccination. On one hand, this could have undermined the population VE if more people chose not to report vaccination with an aim to have shorter interviews. On the other hand, the population VE could have been overestimated if more people reported vaccination because of social desirability bias. However, owing to the comparability of our results to other studies and national data, we believe our results are likely generalisable to the population in Malawi. Similarly, the recall bias could have also affected one of the inclusion criteria where participants who were vaccinated were to confirm if the vaccination was at least 14 days before testing for COVID-19. Research assistants were trained to probe more on this criterion.

As described in the WHO 2021 interim guidance on VE, there is indeed a lack of randomisation of vaccination in real-world settings. Bias would come in as vaccinated persons often differ from unvaccinated persons in their disease risk, independent of vaccination.^33^ We therefore included information on previous COVID-19 infection and other comorbidities in the questionnaire. Apart from not being able to link the available testing data to the vaccination data, we also experienced some erroneous data entries in the national database, which affected the matching process in the study as matching attempts had to be conducted several times; however, data were cleaned rigorously. In cases where data cleaning was not possible, additional matching was carried out and supplemental interviews were conducted to replace the incorrectly completed interviews.

## Conclusion

In this study, we found a low VE of COVID-19 vaccines among community-dwelling individuals in urban, high-burden areas of Malawi. Our findings highlight the need to promote vaccination of the population at large with more emphasis on those at higher risk such as people with chronic underlying illnesses. We recommend the integration of COVID-19 vaccination in routine facility clinics for chronic conditions and continued awareness of the public about the benefits of COVID-19 vaccination and ensuring access to the vaccines. Lastly, national surveillance platforms and health record digitisation are crucial and should be encouraged in managing future pandemics to promote data-driven decision-making by the Ministries of Health.
